# Mechanism of Suppression of Protein Aggregation by α-Crystallin

**DOI:** 10.3390/ijms10031314

**Published:** 2009-03-19

**Authors:** Kira A. Markossian, Igor K. Yudin, Boris I. Kurganov

**Affiliations:** 1 Bach Institute of Biochemistry, Russian Academy of Sciences, Leninsky pr. 33, 119071, Moscow, Russia; 2 Oil and Gas Research Institute, Russian Academy of Sciences, Gubkina st. 3, 117971, Moscow, Russia

**Keywords:** Chaperones, protein aggregation, α-crystallin

## Abstract

This review summarizes experimental data illuminating the mechanism of suppression of heat-induced protein aggregation by α-crystallin, one of the small heat shock proteins. The dynamic light scattering data show that the initial stage of thermal aggregation of proteins is the formation of the initial aggregates involving hundreds of molecules of the denatured protein. Further sticking of the starting aggregates proceeds in a regime of diffusion-limited cluster-cluster aggregation. The protective effect of α-crystallin is due to transition of the aggregation process to the regime of reaction-limited cluster-cluster aggregation, wherein the sticking probability for the colliding particles becomes lower than unity.

## 1. Introduction

α-Crystallin, the major protein of the mammalian eye lens, is present therein as a polydisperse heterooligomer of αA- and αB-crystallins, each of a molecular weight of about 20 kDa [1]. In addition to its structural role in the lens, α-crystallin, as a member of the small heat shock protein (sHSP) family, performs the role of a chaperone and prevents aggregation and precipitation of β- and γ- crystallins (including their self-aggregation) [2–6]. Besides the lens, the αB subunit is expressed extensively in many other tissues, including the brain, lungs, spleen, cardiac and skeletal muscles [7–10], where it functions as a chaperone by interacting with several partially folded target proteins. *In vitro,* α-crystallin (αB- and αA-crystallins) suppresses formation and precipitation of disordered (amorphous) [2,6,11–14], as well as ordered protein aggregates (amyloid fibrils) [15–17].

The quaternary structure of α-crystallin depends on external conditions such as pH, temperature, ionic strength, concentration; the number of its subunits in each assemblage may vary from 15 to 50 [18–22]. Aquilina *et al.* [23] have revealed a distribution, primarily of oligomers containing 24–33 subunits, with the dominant species composed of 28 subunits. Additionally, low levels of oligomers, as small as 10-mers and as large as 40-mers, were observed in αB-crystallin by mass spectrometry. Because of the polydispersity of α-crystallin, the true molecular mass and size of the isolated α-crystallin have not yet been determined and molecular mass values from 300 to 12,000 kDa are variously reported in the literature [24–26]. According to several studies, the quaternary assembly of α-crystallin is characterized by a relatively fast subunit-exchange under physiological conditions, indicating a certain degree of conformational flexibility of the α-crystallin protomers [21,26,27]. This proceeds by monomer exchange among individual oligomers, which is temperature-dependent [21,28]. Heating of α-crystallin at 60 °C results in partial unfolding of the protein, which then doubles in molecular weight and increases in size [28–30]. Evans *et al.* [6] showed that ordered secondary structure of α-crystallin increases with elevated temperatures up to 60 °C, and is rapidly lost at temperatures of 70 °C and above. According to Aquilina *et al.* [31], the rate of subunit exchange is not the critical parameter for chaperone activity of mammalian α-crystallin, in contrast to mechanisms established for analogous proteins from plants, yeast and bacteria.

It is interesting that α-crystallin, like the β- and γ-crystallins, is also able to form fibrils [16,32,33]. Thus, even αA- and αB-crystallins, which are highly stable proteins (like other crystallins), are prone to aggregate and form fibrils, despite their role in protein stabilization in the lens.

α-Crystallin can recognize and bind unfolded proteins over a range of temperatures, and a direct correlation between temperature, monomer exchange and the ability of α-crystallin to suppress amorphous aggregates formation has been demonstrated in a number of studies [11,28,34,35]. Devlin *et al.* [36] showed that α-crystallin specifically inhibits nucleation-dependent aggregation of proteins. This ability has been demonstrated by suppression of aggregation of serine proteinase inhibitors by α-crystallin at 50 °C.

The hydrophobic interaction of α-crystallin with unfolded proteins plays a major role in the recognition of the target protein by the chaperone [34,37,38]. The structural changes of α-crystallin in response to increased temperature support a possible “activation” step (an irreversible increase in ordered secondary structure, associated with assembly into the high-molecular-mass form) in the chaperone mechanism [6,28]. This form of the chaperone associates with partially folded βB_2_-crystallin [6] and α-lactalbumin [39] intermediates prior to protein aggregation. Abgar *et al.* [40] concluded that interaction of α-crystallin with the destabilized protein is a two-step process. First the destabilized proteins are bound by α-crystallin so that non-specific aggregation of the destabilized protein is prevented. This complex is unstable, and a reorganization and inter-particle exchange of the peptides result in stable and soluble large particles.

α-Crystallin was found to prevent formation of amyloid fibrils by various proteins (e.g. amyloid β-peptide, apolipoprotein C-II, α-synuclein, κ-casein) [15,41–43]. The mechanism by which αB-crystallin prevents growth of amyloid fibrils differs from the known mechanism by which it prevents the amorphous aggregation of proteins [42,43]. It has been shown that α-crystallin inhibits formation of amyloid fibrils by Aβ-(1–40) and apolipoprotein C-II due to interaction of the chaperone with the fibril nucleus, and does not inhibit the relatively rapid fibril elongation upon nucleation [42,43]. αB-Crystallin also prevents the fibril growth of β2-microglobulin under acidic conditions [43].

The fibril-suppressing ability of αB-crystallin is temperature dependent, and the nature of the aggregating species with which it interacts is also important [15]. The efficiency of α-crystallin in suppressing fibril formation by κ-casein and α-synuclein increases with temperature. This is consistent with an increased chaperone ability of α-crystallin at higher temperatures to protect target proteins from amorphous aggregation [35]. It has been shown that αB-crystallin interrupted α-synuclein aggregation at its earliest stages, most likely by binding to partially folded monomers and thereby preventing their aggregation into fibrillar structures [15].

The mechanism of interaction of α-crystallin with target proteins has been studied intensively, but details of the mechanism by which α-crystallin accomplishes its chaperone action have not been elucidated.

In the present review a new mechanism of thermal aggregation of globular oligomeric proteins is discussed. According to this mechanism the initial stage of protein aggregation is the stage of formation of the initial aggregates. The starting aggregates contain hundreds of denatured protein molecules. Further growth of protein aggregates is accomplished as a result of the sticking of the starting aggregates or the aggregates of higher order. This stage of protein aggregation proceeds in the diffusion-limited cluster-cluster aggregation (DLCA) regime. This regime implies that each collision results in the sticking of the interacting particles. In the frame of this mechanism of protein aggregation the protective action of α-crystallin is due to diminution of the size of the start aggregates and transition of the aggregation process from the DLCA regime to the reaction-limited cluster-cluster aggregation (RLCA) regime wherein the sticking probability for the colliding particles becomes lower than unity.

## 2. Mechanisms of Protein Aggregation

One of the earliest studies of protein aggregation was that by London *et al*. [44], who revealed an intermediate of the enzyme tryptophanase that aggregated after exposure to 8 M urea. More recently, the idea that partially folded intermediates might be responsible for protein aggregation has evolved [45–54]. Native proteins can unfold under the action of denaturing agents such as guanidine hydrochloride or urea, as well as due to thermal denaturation. Inappropriate exposure of hydrophobic sequences of unfolded proteins leads to formation of non-native conformations, which can interact with existing aggregates [55]. The aggregates can be either amorphous structures, such as inclusion bodies [49,56–58], or ordered fibrils such as amyloid plaques and prion particles [55,59,60].

A distinctive characteristic of the kinetic curves of protein aggregation is the existence of an initial lag period. To explain such a peculiarity of protein aggregation, some models including a nucleation stage were proposed. These models are based on the nucleation-dependent polymerization model developed by Oosawa and Kasai [61]. The reversible association of proteins involves the stage of formation of a nucleus, further growth of the associate proceeding by attachment of monomer to the growing associate. By analogy with the nucleation-dependent polymerization model, a nucleation-dependent aggregation model explaining the peculiarities of the kinetics of irreversible aggregation of proteins has been proposed [62–69]. The model of nucleation-dependent aggregation can be formulated as follows. The first step is unfolding of the protein molecule:

(1) N→U,

where N and U are the native and unfolded states of the protein molecule. Association of several molecules U results in formation of a nucleus:

(2) $$n\text{U}\to {\text{U}}_{n},$$

where U*_n_* is a nucleus consisting of *n* monomers U. The stage of aggregate growth is expressed in the attachment of monomer U to the nucleus or aggregates of higher order:

(3) $${\text{U}}_{n}\overset{+\text{U}}{\to }{\text{U}}_{n+1}\overset{+\text{U}}{\to }{\text{U}}_{n+2}\overset{+\text{U}}{\to }\ldots \overset{+\text{U}}{\to }{\text{U}}_{m}.$$

When all monomer U is exhausted, the aggregate of a limiting size U*_m_*, consisting of *m* monomers U, is formed. Theoretical analysis of the kinetics of nucleation-dependent aggregation has been performed by Kodaka [68].

Ben-Zvi and Goloubinoff [70] showed that sub-stoichiometric amounts of one fast co-aggregating protein can significantly increase thermal aggregation kinetics of otherwise soluble, slow-aggregating protein. The ability of aggregates to seed and propagate aggregation of other proteins is consistent with the idea that thermal aggregation of proteins includes the stage of nucleation.

A number of works are devoted to models of nucleation-dependent aggregation describing amyloid fibril formation [71–77]. Generation of oligomeric aggregation nuclei is considered as a key step at the onset of protein aggregation, accounting for the delay times of polymer appearance that are recorded by *in vitro* protein aggregation experiments. It has been shown that in the course of immunoglobulin light chain aggregation a relatively unfolded, but compact intermediate (characterized by decreased tertiary and secondary structure at pH below 3) leads to rapid formation of amyloid fibrils. The native-like intermediates, observed between pH 4 and 6, with little loss of secondary structure, but with significant tertiary structure changes, predominantly result in formation of amorphous aggregates [77]. Amyloid fibril formation involves the ordered self-assembly of partially folded species, which are critical soluble precursors of fibrils [78]. It is also indicated that fibril elongation involves addition of a partially unfolded intermediate, rather than a protein in the native state [79]. Kumar *et al.* [80] have studied amyloid fibril formation by the small protein barstar. Barstar forms a stable soluble oligomer at low pH, which can transform into protofibrils. The absence of a lag phase suggests that aggregation of stable soluble oligomer forms into protofibrils is not nucleation-dependent. The rate of aggregation increases with increasing protein concentration, but reaches the limit at high concentrations. An analysis of the dependence of the apparent rates of protofibril formation indicates that the slowest stage in protofibil formation is lateral association of linear aggregates. Overall, the study indicates that growth in the course of protofibril formation occurs step-wise through progressively larger and larger aggregates, via multiple pathways, and finally through lateral association of critical aggregates.

Since protein aggregates possess a higher light scattering ability than the original molecules, a convenient way to study protein aggregation kinetics is registration of the increment of the light scattering intensity or apparent optical absorbance. Some authors have tried to analyze the kinetic curves of protein aggregation assuming that proportionality between the apparent optical absorbance and the amount of the aggregated protein exists (for example, [65,66,81]). However it is clear that more reliable information on the mechanism of protein aggregation may be obtained using methods that allow sizing the protein aggregates. On-line analysis of the kinetics of protein aggregation can be carried out using dynamic light scattering (DLS). There are numerous investigations of the aggregation kinetics for different proteins, where the size of protein aggregates was estimated by DLS [14,52,80,82–89].

DLS is commonly used to determine the size of nanoparticles in a suspension by measuring the dynamics of the light-scattering intensity fluctuations. Colloidal particles or macromolecules suspended in fluid undergo Brownian motion. This motion causes fluctuations in the local concentration of the particles resulting in local non-homogeneities of the refractive index. This in turn leads to the light scattering phenomenon. The measured time-dependent autocorrelation function G2(τ) of the scattered light is a function of the delay time τ and for a single decay rate can be represented by the following equation:

(4) $$G_{2}(\tau )=A[1+B\;\text{exp}\;(-2\Gamma \tau )].$$

This function describes light scattering in system of monodisperse spherical non-interacting particles. The time-independent baseline correlation level *A* is proportional to the square of the time-averaged intensity *I*. The factor *B* is called usually as the intercept of the autocorrelation function. This instrument factor is a measure of quality of light scattering conversion, which is possible to define as the relative coherence of the received scattered light. In accordance with definition *B* ≤ 1, practical value of *B* within 0.2–0.9 is acceptable for precise measurement of particle size.

The decay rate Γ of the time-dependent correlation function is directly related to the translation diffusion coefficient *D* of the particles:

(5) $$\Gamma =D_{q}^{2},$$

where *q* is the modulus of the scattering vector ( $q=\frac{4\pi \text{n}}{\lambda_{0}}\text{sin}\frac{\theta }{2}$), *n* is the refractive index of the solvent, λ_0_ is the wavelength of the incident light in vacuum and θ is the scattering angle.

The mean hydrodynamic radius of the particles, *R*_h_*,* then can be calculated using the Stokes-Einstein equation:

(6) $$D=\frac{k_{\text{B}}T}{6\pi \eta R_{\text{h}}},$$

where *k*_B_ is Boltzmann’s constant, *T* is the absolute temperature and η is the viscosity of the solvent. The size calculated from Equation (6) is called the hydrodynamic radius. It may be larger than the radius of the bare particles because of possible layers of solvent, surfactant molecules or (for charged particles) adsorbed ions. In most cases these layers add a negligible correction to the size, except for the smallest sizes measurable. Eqs. (4) and (5) are valid for noninteracting spherical particles. If the particles are involved in an aggregation process, they certainly interact. However, these equations are still applicable to monitor the change of the apparent (“effective”) particle size if the characteristic time of aggregation kinetics is much larger than the time of the measurements.

Direct fitting of experimental data by Equation (4) allows the effective average decay rate to be found. However in experimental practice it is important to know the polydispersity of the particles. Usually, light scattering in polydisperse multicomponent samples gives a multiexponential correlation function. The simple approximation with an appropriate multiexponent model is not effective due to serious mathematical complexity for solution of such, so-called, ill-posed problem. A few methods have suggested solving this problem. The most popular and simple is method of cumulants, which is based on cumulant expansion of the correlation function within average decay rate [90,91]. A final cumulant expression, suitable for fitting practice, is:

(7) $$G_{2}(\tau )=A\left[1+B\;\text{exp}(-2\bar{\Gamma }\tau ){\left(1+\frac{\mu_{2}}{2!}\tau^{2}-\frac{\mu_{3}}{3!}\tau^{3}\right)}^{2}\right],$$

where Γ̅ is the average decay rate, μ_2_ and μ_3_ are the second and third cumulants. Cumulants μ_2_ and μ_3_ allow the parameters of a polydispersity distribution to be calculated: polydispersity index PI = μ_2_/Γ̅^2^ characterizing the dimensionless width of the polydispersity distribution (see ISO standard [92]) and asymmetry index (skewness) AI = μ_2_/μ_3_; Γ̅ characterizing the dimensionless asymmetry of the polydispersity distribution. Unfortunately, the most mathematically-rigorous method of cumulants can be used only for the unimodal relatively narrow particle size distributions. More universal method of the DLS data processing is based on the regularized Laplace inversion. This method allows finding polymodal particle size distributions with satisfactory reliability of fitting. There are a few such programs. The most popular CONTIN software was developed by S.W. Provencher many years ago. A new version of the regulation software DYNALS has been developed by A. Goldin (Alango, Israel; http://www.photocor.com/dynals.htm). The DYNALS package is well-proven and used by many researchers.

Features of the Photocor Complex DLS setup (Photocor Instruments Inc., USA) are typical in multipurpose installations for studying both static (intensity) and dynamic (time-dependent correlation function) light scattering [93]. The optical bench and precision goniometer are based on a massive base. An He-Ne laser (Coherent, USA, Model 31-2082, 632.8 nm, 10 mW) and the focusing system are fixed on the bench. The thermostat and cell holder are coaxial with the goniometer axis. The temperature is controlled by the PID temperature controller Photocor-TC to within ±0.1 °C. The receiving system (photon counting system Photocor-PC2) is located on the rotating goniometer arm. The photon counting system consists of a set of pinholes for selection of aperture, a low-noise photomultiplier tube (PMT) and an amplifier-discriminator. To avoid the afterpulsing distortions the quasi-cross correlation scheme with two PMTs is used. The output signal produced by the photon counting system occurs as a sequence of standard pulses corresponding to escaped photoelectrons. The raw signal from the photon counting system is analyzed by a single-board correlator Photocor-FC (http://www.photocor.com), which is plugged into a personal computer. The correlator is a multichannel device that performs real-time data processing and accumulation of the correlation function of the light scattering fluctuations. The correlator Photocor-FC has two time-scale modes of operation, i.e., a linear mode with equidistant points of the measured correlation function and a so called multiple-tau mode with the logarithmic order of the correlation points in time. Operation of the correlator in multiple-tau mode needs no tuning of time-scale and is suitable for measurements of multi-peak particle distributions. The linear scale of the correlator enables one to achieve maximum accuracy for measurements of a mono-modal narrow distribution. These cross-correlation system and fast correlator allow measuring particle size in very wide range of 1 nm to 5 μm. The personal computer performs data analysis and instrument control.

**Figure 1.**
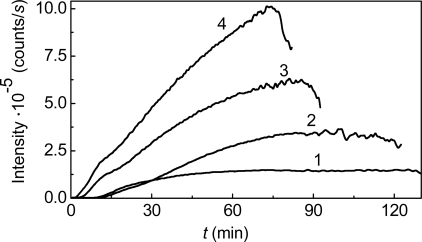
Kinetics of thermal aggregation of GAPDH (0.4 mg/mL) at 55 **°**C (10 mM Na-phosphate buffer, pH 7.5). The dependences of the light scattering intensity on time obtained at various concentrations of the enzyme: (1) 0.05, (2) 0.2, (3) 0.3 and (4) 0.4 mg/mL.

Consider, as an example, the kinetics of thermal aggregation of glyceraldehyde-3-phosphate dehydrogenase (GAPDH) from rabbit skeletal muscle. Figure 1 shows the dependences of the light scattering intensity on time for GAPDH aggregation at 55 °C. These dependences were obtained at various concentrations of the protein in the interval from 0.05 to 0.4 mg/mL. Diminution of the light scattering intensity at rather high values of time is due to precipitation of large-sized protein aggregates.

**Figure 2.**
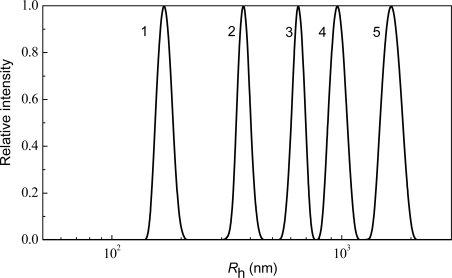
Analysis of thermal aggregation of GAPDH (0.4 mg/mL) at 55 C by DLS. Distribution of the protein aggregates by size for aggregation of GAPDH at varioustimes of incubation: (1) 5, (2) 10, (3) 20 (4) 30 and (5) 70 min.

**Figure 3.**
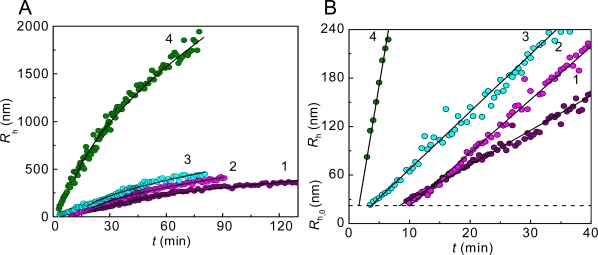
Kinetics of thermal aggregation of GAPDH (0.4 mg/mL) at 55 °C. The dependences of the hydrodynamic radius of the protein aggregates (*R*_h_) on time obtained at various concentrations of the enzyme: (1) 0.05, (2) 0.2, (3) 0.3 and (4) 0.4 mg/mL. (A) Full kinetic curves. The solid curves are calculated from Equation (8). (B) The initial parts of the dependences of *R*_h_ on time. The solid lines were calculated from Equation (9). The horizontal dotted line corresponds to the *R*_h,0_ value (*R*_h,0_ = 22 nm).

According to the DLS data, the distribution of protein aggregates by size is unimodal, with the position of a maximum shifted towards the higher values of the hydrodynamic radius with increasing incubation time (Figure 2). Of special interest are the dependences of the hydrodynamic radius (*R*_h_) of the protein aggregates on time (Figure 3).

It was important to elucidate whether the dependences of *R*_h_ on time approached the limiting value at high values of time. The analysis carried out for thermal aggregation of different proteins [14,94–97] showed that above a definite point of time (*t* > *t* *) the dependences of *R*_h_ on time followed the power law:

(8) $$R_{\text{h}}=R_{\text{h}}^{*}{\left[1+K_{2}(t-t^{*})\right]}^{1/d_{\text{f}}},$$

where $R_{\text{h}}^{*}$ is the value of *R*_h_ at *t* = t*, *K*_2_ is a constant and *d*_f_ is the fractal dimension of the protein aggregates. The fractal dimension is a structural characteristic of aggregates which are formed as a result of unordered interactions (random aggregation). The mass of an aggregate (*M*) formed in such a way is connected with its effective radius (*R*) by the following relationship: *M* ~ *R*^*d*_f_^. The value of *d*_f_ for GAPDH aggregation (Figure 3A) was found to be 1.79 ± 0.05. Such dependences of *R*_h_ on time coincide with the DLCA regime of aggregation [98–102]. Under this kinetic regime of aggregation each collision of protein clusters results in their sticking. The DLCA regime is characterized by the universal value of *d*_f_ (*d*_f_ ≈ 1.8) [98,103].

It is evidenced by Figure 3B that the initial parts of the dependences of the hydrodynamic radius of protein aggregates on time are linear. It should be noted that such dependences of hydrodynamic radius on time (initial linear parts followed by the parts described by power law) are typical of aggregation of colloids proceeding in the DLCA regime (see, for example, [108]. The linear part of the dependence of *R*_h_ on time can be described by the following equation [95]:

(9) $$R_{\text{h}}=R_{\text{h},0}[1+\frac{1}{t_{2\text{R}}}(t-t_{0})],$$

where *R*_h,0_ is the hydrodynamic radius of the start aggregates, *t*_0_ is the point of time when the start aggregates appear, and *t*_2R_ is the time interval over which the hydrodynamic radius of the protein aggregates increases from *R*_h,0_ to 2*R*_h,0_. Parameter *t*_2R_ characterizes the rate of aggregation. The higher the 1/*t*_2R_ value, the higher is the aggregation rate. Knowing the *R*_h,0_ value, we can calculate parameters *t*_0_ and *t*_2R_. In the case of thermal aggregation of GAPDH (Figure 3B) the increase in protein concentration resulted in diminution of the lag period characterized by parameter *t*_0_ and the increase in the aggregation rate characterized by the 1/*t*_2R_ value.

Analysis of the light scattering intensity versus the hydrodynamic radius of the protein aggregates plots lead to an important conclusion about the mechanism of protein thermal aggregation.

**Figure 4.**
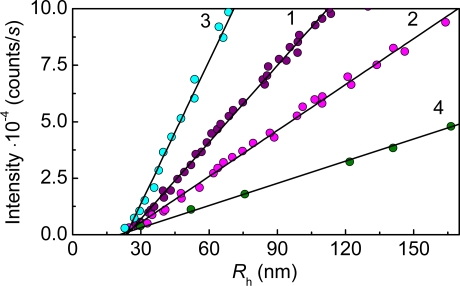
Kinetics of thermal aggregation of GAPDH (0.4 mg/mL) at 55 °C. Light scattering intensity versus *R*_h_ plots constructed for various concentrations of the enzyme: (1) 0.05, (2) 0.2, (3) 0.3 and (4) 0.4 mg/mL.

As is seen from Figure 4, the protein aggregates of rather large size are present in the system at the initial moment of the increment of the light scattering intensity (*I*). These initial aggregates were called “starting aggregates” [95]. The hydrodynamic radius of the starting aggregates (*R*_h,0_) corresponds to the length cut off on the abscissa axis by the linear dependence of *I* on *R*_h_. *R*_h,0_ remained practically constant at variation of the GAPDH concentration in the interval from 0.05 to 0.4 mg/mL and was found to be 22 ± 1nm (Figure 4).

In the framework of the proposed mechanism of protein aggregation it becomes clear why the dependences of the light scattering intensity on time tend to level off at rather high values of time. The light scattering ability of the protein aggregate increases with the increase in the aggregate size. However diminution of particle concentration takes place because of their sticking. As a result we observe an apparent leveling off for the light scattering intensity versus time plots. It is significant that this “leveling off does not indicate the completion of the aggregation process. If we measure the size of protein aggregates on the “plateau”, we shall find that the growth of aggregates continues.

**Figure 5.**
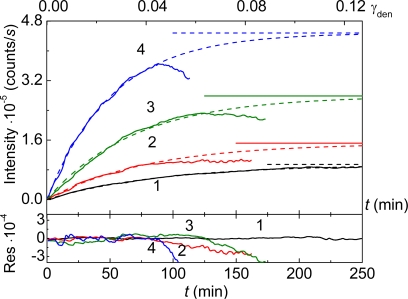
Thermal aggregation of mAAT (10 mM Na-phosphate buffer, pH 7.5) at 60 °C. Dependences of the light scattering intensity on time obtained at various concentrations of protein: (1) 0.05, (2) 0.1, (3) 0.2 and (4) 0.4 mg/mL. The upper scale corresponds to the portion of the denatured protein (γ_den_) calculated from the DSC data. Dotted curves are calculated from Equation (8). The horizontal dotted lines correspond to the *I*_lim_ values. The lower panel shows the residuals (Res).

Thermal aggregation of aspartate aminotransferase from pig heart mitochondria (mAAT) may serve as another example demonstrating the importance of the light scattering intensity versus the hydrodynamic radius plots and supporting the idea of the start aggregate formation as an initial stage of protein aggregation [109]. Figure 5 shows the dependences of the light scattering intensity on time for mAAT aggregation at 60 °C. The dependences were obtained at various concentrations of mAAT in the interval from 0.05 to 0.4 mg/mL.

The ascending part of the dependence of the light scattering intensity (*I*) on time (before the precipitation of large-sized aggregates) obeys the exponential law [65]:

(10) $$I=I_{\text{lim}}[1-\text{exp}(-k_{\text{I}}t)],$$

where *I*_lim_ is the limiting value of *I* at *t* → ∞ and *k*_I_ is the rate constant of the first order. The upper abscissa axis (Figure 5) shows the portion of the denatured protein (γ_den_) calculated from the data of differential scanning calorimetry (DSC). As has been shown by Golub *et al*. [110], the portion of the denatured protein is identical to the portion of the aggregated protein calculated from measurements of the optical absorbance of the heated protein solution after 30 min centrifugation at 20,000 g. The analysis of the data presented in Figure 5 using Equation (10) shows that the increment of the light scattering intensity is terminated even before the portion of the denatured protein reaches 22% [109].

**Figure 6.**
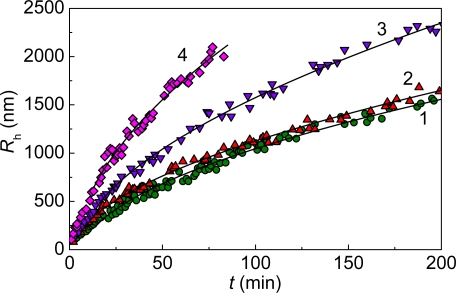
Dependences of the hydrodynamic radius (*R*_h_) of the protein aggregates on time for aggregation of mAAT at 60 **°**C obtained at various protein concentrations: (1) 0.05, (2) 0.1, (3) 0.2 and (4) 0.4 mg/mL. Solid curves were calculated in accordance with Equation (9).

mAAT aggregation is accompanied by the growth of protein aggregates (Figure 6). As in the case of GAPDH thermal aggregation, the dependences of *R*_h_ on time involve the initial linear parts followed by the parts which are described by power law (Equation 8) with *d*_f_ = 1.82 ± 0.02. The value of *d*_f_ (the fractal dimension of the aggregates) indicates that mAAT aggregation proceeds in the DLCA regime.

**Figure 7.**
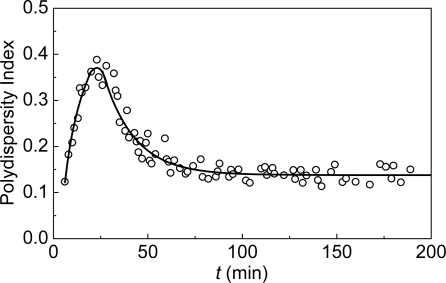
Dependence of the polydispersity index PI calculated according with International standard ISO 22412:2008(E) on time for aggregation of mAAT (0.2 mg/mL) at 60 °C.

The width of the size distribution of protein aggregates formed in the course of mAAT aggregation may be characterized by the polydispersity index (PI) [92]. The temporal character of the changes in the PI value (Figure 7) shows that two parts of the dependences of *R*_h_ on time correspond to different physical processes proceeding in the system. The increase in the *R*_h_ value over the initial linear part of the dependence of *R*_h_ on time is accompanied by the increase in the PI value. The broadening of the distribution of the protein aggregates by size in this time interval is due to the fact that two processes, namely, formation of new start aggregates and their sticking, proceed simultaneously. At *t* > t* the prevailing process is the sticking of the protein aggregates accompanied by the decrease in the PI value. The limiting value of PI at *t* → ∞ was found to be 0.14 ± 0.01. Such a low PI value is typical of aggregation of colloids proceeding in the DLCA regime [98].

Analysis of the dependence of the hydrodynamic radius of the protein aggregates on time is of considerable importance for understanding the mechanism of protein aggregation. As discussed above, in the case of thermal aggregation of GAPDH and mAAT, dependences of *R*_h_ on time follow the power law with *d*_f_ parameter close to 1.8. A similar picture was observed for thermal aggregation of β_L_-crystallin from bovine eye lens [95], tobacco mosaic virus coat protein [96] and glycogen phosphorylase *b* (Ph*b*) from rabbit skeletal muscle [97,111]. Thus one can assume that thermal aggregation of proteins generally proceeds in the DLCA regime.

**Figure 8.**
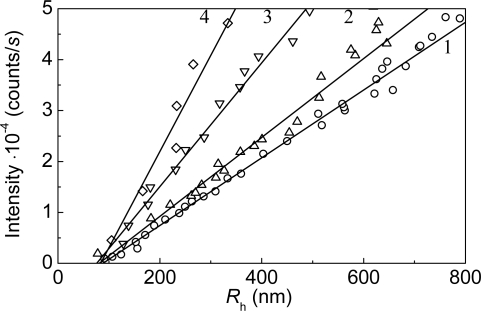
Relationship between the light scattering intensity and hydrodynamic radius of the protein aggregates for aggregation of mAAT at 60 °C. The protein concentrations were as follows: (1) 0.05, (2) 0.1, (3) 0.2 and (4) 0.4 mg/mL.

Construction of the light scattering intensity versus the hydrodynamic radius plots allowed sizing the start aggregates formed in the course of thermal aggregation of mAAT (Figure 8). The hydrodynamic radius of the start aggregates (*R*_h,0_) may be calculated from the length cut off on the abscissa axis by the linear dependence of *I* on *R*_h_. The *R*_h,0_ value was found to be 79 ± 3 nm. It should be noted that the hydrodynamic radius of the native dimeric molecule of mAAT with molecular mass of 91.2 kDa under non-denaturing conditions is 4.13 ± 0.18 nm. A rough estimate of the number of unfolded mAAT monomers in the start aggregate gives the value of 1030 ± 100.

The light scattering intensity versus the hydrodynamic radius plots were also used for estimation of the start aggregate size for thermal aggregation of β_L_-crystallin from bovine eye lens [95], tobacco mosaic virus coat protein [96] and Ph*b* [112].

**Figure 9.**
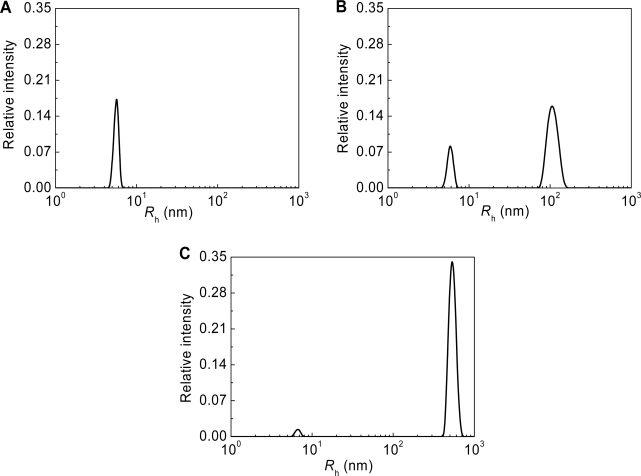
Thermal aggregation of GAPDH (1 mg/mL) at 45 **°**C (10 mM Na-phosphate buffer, pH 7.5). Distribution of the particles by size registered at various times of incubation: (A) 3.0, (B) 5.5 and (C) 17.0 min.

**Figure 10.**
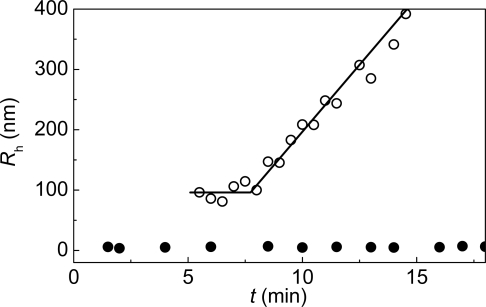
Dependences of the hydrodynamic radius (*R*_h_) for the original GAPDH (solid circles) and protein aggregates (open circles) on time for aggregation of GAPDH (1 mg/mL) at 45 **°**C (10 mM Na-phosphate buffer, pH 7.5). The concentration of GAPDH was 1 mg/mL.

To obtain more reliable information on the stage of the starting aggregate formation, conditions had to be chosen, whereby the simultaneous registration of the native enzyme and starting aggregates was possible. For this purpose aggregation of GAPDH (1 mg/mL) at 45 °C was studied [113]. The time of accumulation of the autocorrelation function was 30 s. Over first four minutes only the native GAPDH tetramer with *R*_h_ = 5.4 ± 0.3 nm was detected (Figure 8A). At *t* = 5 min the starting aggregates with *R*_h_ = 97.0 ± 0.2 nm appeared. Over the interval from 5 to 8 minutes the size of the protein aggregates remained constant. At *t* > 8 min the size of the protein aggregates began to increase because of the sticking of the starting aggregates (Figure 9B and C and Figure 10). These data demonstrate that formation of the starting aggregates proceeds on the all-or-none principle without accumulation of intermediates.

Sluzky *et al*. [114] were the first to observe formation of primary protein aggregates with the hydrodynamic radius of 85 ± 10 nm, when studying insulin aggregation upon shaking at 37 °C. When studying the thermal aggregation of β-lactoglobulin at pH 7.0 by means of size exclusion chromatography, it was shown that there was a clear separation between a narrow peak that corresponded to residual native protein and a broad peak that corresponded to the aggregates [86,115–117]. This observation implied that the aggregates of the minimum size contained many monomers, and that no or very few stable oligomers were formed. In accordance with these data, the authors supposed that the first stage of thermal aggregation of β-lactoglobulin is the step of primary aggregate formation. Further association of primary aggregates gives large self-similar aggregates with fractal dimension *d*_f_ of 1.7 in the absence of salt [117]. Although the primary aggregates postulated in the noted works [86,115–118] are cross-linked by intermolecular disulfide bonds (in pH range 6.4-8.0), such aggregates are evidently similar to the start aggregates we observed for β_L_-crystallin and other proteins [14,95].

On the basis of the data of Durand *et al*. [118] on the sensitivity of sticking of the primary aggregates to variation of ionic strength, one can assume that the starting aggregates may be sufficiently stable at low ionic strength values. From this point of view it is of interest to discuss the properties of the so-called inactivated actin. Turoverov *et al*. [119] observed that 60 min incubation of rabbit skeletal muscle actin (5 mM Tris-HCl, pH 8.2) at 60 °C resulted in the formation of relatively stable particles with average sedimentation coefficient of 20 S (inactivated actin). The size of particles of inactivated actin was independent of the protein concentration (within the limits 0.05–1.0 mg/mL). A dramatic increase in 1-anilinonaphthalene-8-sulfonate binding to inactivated actin, in comparison with native and unfolded protein, indicated that the inactivated actin had solvent-exposed hydrophobic clusters on the surface. In our opinion inactivated actin may be considered as a “frozen” starting aggregate.

**Figure 11.**
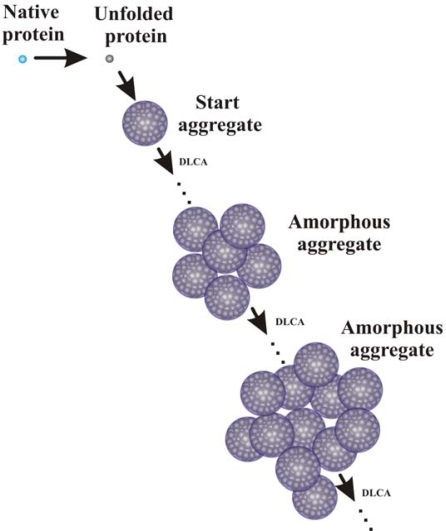
Mechanism of heat-induced aggregation of proteins resulting in formation of the amorphous aggregates.

Thus, investigation of the kinetics of thermal aggregation of proteins by DLS allowed proposing the mechanism of protein aggregation demonstrated in Figure 11 [14,95]. The initial stage of the aggregation process is formation of the starting aggregates. Further sticking of the starting aggregates and aggregates of higher order proceeds in the DLCA regime.

## 3. Mechanism of Protein Aggregation in the Presence of α-crystallin

**Figure 12.**
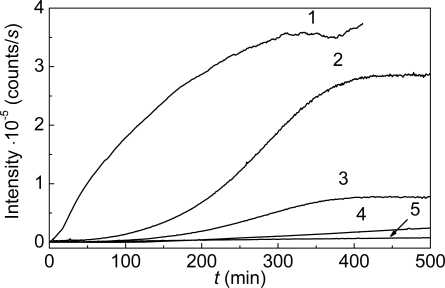
Suppression of heat-induced aggregation of mAAT by α-crystallin. The dependences of the light scattering intensity on time for aggregation of mAAT (0.2 mg/mL or 4.4 μM in calculation to monomer) at 60 **°**C (10 mM Na-phosphate buffer, pH 7.5). The concentrations of α-crystallin were as follows: (1) 0, (2) 0.1, (3) 0.15, (4) 0.2 and (5) 0.4 mg/mL or (1) 0, (2) 5, (3) 7.5, (4) 10 and (5) 20 μM (in terms of monomer).

Suppression of thermal aggregation of proteins by α-crystallin is a well-studied phenomenon [2,3,11,28,34,35]. Figure 12 shows the effect of α-crystallin on aggregation of mAAT at 60 °C [109]. The addition of α-crystallin results in a lower increment of the light scattering intensity in time.

**Figure 13.**
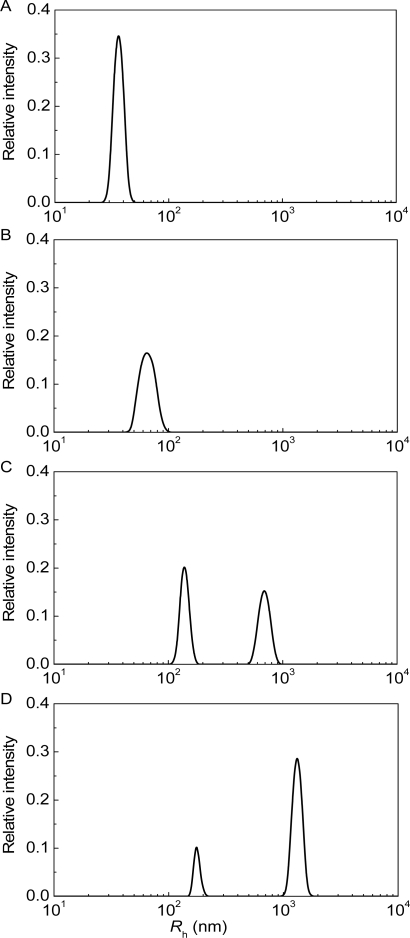
Distribution of the protein aggregates by size for aggregation of mAAT (0.2 mg/mL) at 60 **°**C in the presence of α-crystallin (0.2 mg/mL). The values of the incubation time were as follows: (A) 100, (B) 200, (C) 300 and (D) 350 min.

DLS made it possible to characterize the change in the distribution of the protein aggregates by size in the course of mAAT aggregation in the presence of α-crystallin [109]. At low times of incubation the distribution of protein aggregates by size was unimodal (Figure 13A and B; incubation times were 100 and 200 min). However, the splitting of protein aggregate population into two components took place in the course of the aggregation process (Figure 13C and D; incubation times were 300 and 350 min).

It should be noted that bimodal character of distribution of the protein aggregates at rather high values of the incubation time was also observed for thermal aggregation of β_L_-crystallin from bovine eye lens [95] and GAPDH from rabbit skeletal muscle [14].

**Figure 14.**
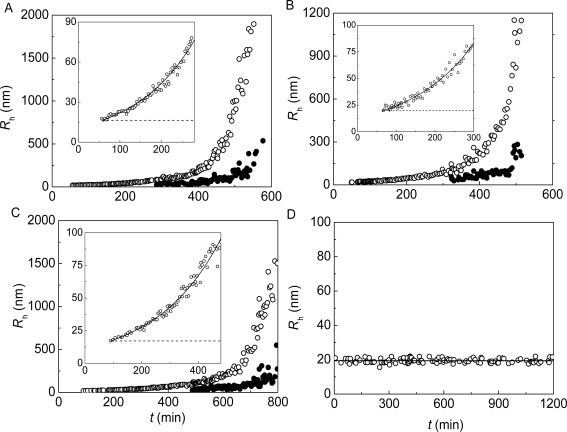
Dependences of hydrodynamic radius (*R*_h_) of the protein aggregates on time for aggregation of mAAT (0.2 mg/mL) at 60 **°**C in the presence of α-crystallin. The concentrations of α-crystallin were as follows: (A) 0.1, (B) 0.15, (C) 0.2 and (D) 0.4 mg/mL. Insets in panels A–C show the initial parts of the dependences of *R*_h_ on time. Solid curves are calculated from Equation (11). The dotted horizontal lines correspond to the *R*_h,0_ values. At rather high values of time (*t* > *t*_crit_) the population of aggregates is split into two components, which are designated by open and solid circles (panels A–C).

The distinctive property of the dependences of the protein aggregate hydrodynamic radius on time for mAAT aggregation in the presence of α-crystallin is that the initial parts of these dependences follow the exponential law:

(11) $$R_{\text{h}}=R_{\text{h},0}\left\{\text{exp}\left[\frac{\text{ln}\;2}{t_{2\text{R}}}(t-t_{0})\right]\right\},$$

where *R*_h,0_ is the hydrodynamic radius of the initial particles participating in the aggregation process, *t*_0_ is the duration of the lag period for the aggregation process and *t*_2_*_R_* is the time interval over which the *R*_h_ value of aggregates is doubled. Parameter *t*_2_*_R_* characterizes the rate of aggregation. The higher 1/*t*_2_*_R_* value, the higher is the rate of aggregation. If parameter *R*_h,0_ is known, parameter *t*_0_ may be calculated using Equation (11). The solid curves in the insets in Figure 14A-C are calculated from Equation (11). The 1/*t*_2_*_R_* value decreased from 1.0 to 0.07 min^−1^, when the concentration of α-crystallin increased from 0.15 to 0.4 mg/mL.

The exponential character of the dependence of the hydrodynamic radius of the protein aggregates on time indicates that the aggregation process proceeds in the regime of reaction-limited cluster-cluster aggregation (RLCA) [98,101,108,120]. Fulfillment of the RLCA regime means that the sticking probability for the colliding particles is lower than unity. It should be noted that the exponential character of the dependences of the hydrodynamic radius of the protein aggregates on time was also observed for thermal aggregation of β_L_-crystallin [95], GAPDH [14] and Ph*b* [97] studied in the presence of α-crystallin.

Figure 14 shows the dependences of the hydrodynamic radius of the protein aggregates on time obtained for aggregation of mAAT (0.2 mg/mL) in the presence of various concentrations of α-crystallin in the interval from 0.1 to 0.4 mg/mL [109]. As is evident from this Figure, a clearly expressed splitting of the protein aggregate population into two components at rather high values of incubation time was observed at the α-crystallin concentrations of 0.1, 0.15 and 0.2 mg/mL (panels A, B and C). The point of time when such a splitting occurred was designated as *t*_crit_ [95]. At α-crystallin concentration of 0.4 mg/mL, the growth of protein aggregates was strongly suppressed.

It should be noted that bimodal character of distribution of the protein aggregates at rather high values of the incubation time was also observed for thermal aggregation of β_L_-crystallin from bovine eye lens [95] and GAPDH from rabbit skeletal muscle [14].

**Figure 15.**
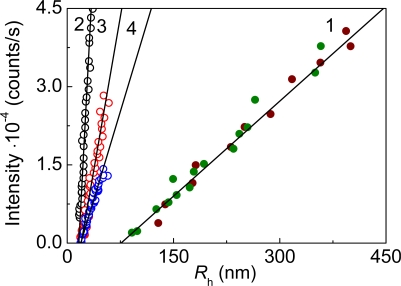
Relationship between the light scattering intensity and hydrodynamic radius of the protein aggregates for aggregation of mAAT (0.2 mg/mL) at 60 **°**C in the presence of the following concentrations of α-crystallin: (1) 0, (2) 0.1, (3) 0.15, (4) 0.2 and (5) 0.4 mg/mL.

The light scattering intensity versus the hydrodynamic radius plots may be used for estimation of the hydrodynamic radius of the initial particles (*R*_h,0_) participating in the aggregation process. As it is demonstrated in Figure 15, the *R*_h,0_ values measured for mAAT aggregation in the presence of α-crystallin at concentrations of 0.1, 0.15 and 0.2 mg/mL were practically identical. The average value of the *R*_h,0_ was found to be 17.7 ± 1.3 nm. It should be noted that this *R*_h,0_ value exceeds the *R*_h,0_ value for α-crystallin under non-aggregating conditions (*R*_h_ = 11.0 ± 0.1 nm) [121]. The hydrodynamic radius of the start aggregates was found to be 79 ± 3 nm in the case of thermal aggregation of mAAT in the absence of α-crystallin. Thus drastic decrease in the size of the start aggregates takes place in the presence of α-crystallin. An analogous picture was observed when studying the effect of α-crystallin on thermal aggregation of β_L_-crystallin [95], GAPDH [14] and Ph*b* [112].

The reason of the splitting of the protein aggregate population into two components in the presence of α-crystallin may be the following. When the process of protein aggregation is studied in the absence of the chaperone, aggregation proceeds in the DLCA regime. Fulfillment of this regime means that each collision of the interacting particles results in their sticking. The high sticking probability is ensured by the high density of sticking sites (the sites of aggregation) on the surface of a starting aggregate. When aggregation of the protein substrate proceeds in the presence of α-crystallin, the starting aggregates containing incorporated α-crystallin are formed. Such starting aggregates are characterized by a lower density of the aggregation sites on the aggregate surface. As a result, the sticking probability for such colliding particles becomes lower than unity. Consider the sticking of particles with a limited number of the aggregation sites on the particle surface. The sticking of two particles results in disappearance of the aggregation sites in the contact region because of their mutual screening. Thus the growth of the protein aggregate may bring about formation of aggregates in which the number of the aggregation sites on the aggregate surface is greatly reduced. Such aggregates correspond to the component with lower values of *R*_h_ in the aggregate population (solid circles in Figure 14A–C at *t* > *t*_crit_). From this point of view the component with higher values of *R*_h_ (hollow circles in Figure 14A–C at *t* > *t*_crit_) corresponds to the component that retains relatively high density of the aggregation sites on the aggregate surface [95].

**Figure 16.**
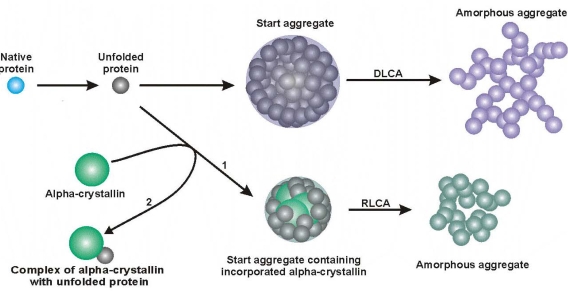
Mechanism of heat-induced aggregation of proteins in the presence of α-crystallin. Pathways 1 and 2 show aggregation of the protein substrate at relatively low and relatively high concentrations of α-crystallin, respectively.

The study of the effect of α-crystallin on thermal aggregation of proteins by DLS shows that the protective action of α-crystallin is due to diminution of the size of the start aggregates, transition of the aggregation process from the DLCA regime to the RLCA regime and, consequently, the decrease in sticking probability for the colliding particles. At rather high concentrations of α-crystallin, for example, at concentration of 0.4 mg/mL for the conditions used in the experiments shown in Figure 14, α-crystallin -unfolded protein complexes are formed, which are incapable of aggregating. The overall mechanism of the protective action of α-crystallin is represented in Figure 16 [14,95].

Since the addition of α-crystallin results in the transition of the aggregation process for the protein substrate from the DLCA regime to the RLCA regime, one can expect that aggregation of α-crystallin itself will proceed in the RLCA regime. Actually Andreasi Bassi *et al.* [82] showed that the dependence of the hydrodynamic radius on time for the protein aggregates formed in the course of aggregation of α-crystallin at 55 °C in the presence of 16 mM CaCl_2_ (10 mM Tris-HCl buffer, pH 7.4) followed the exponential law.

## 4. Effect of α-Crystallin on Thermal Stability of Proteins

In this review we have discussed the effect of α-crystallin on heat-induced aggregation of proteins. Since the initial stage of thermal aggregation of proteins is unfolding of the protein molecule, it is of interest to discuss the effect of α-crystallin on protein stability. The analysis of literature data shows that α-crystallin usually does not affect thermal stability of the proteins or, in some instances, stabilizes the proteins. For example, it was shown that α-crystallin interfered with heat-induced inactivation of pig heart citrate synthase, cytosolic malate dehydrogenase [122], catalase [122,123], sorbitol dehydrogenase from sheep liver [124], the restriction enzyme Nde I cleaving plasmid DNA [125] and *Chlamydia pneumoniae* uracil DNA glycosylase [126]. Rajaraman *et al.* [122] have concluded that interaction of α-crystallin with early unfolded protein intermediates reduces the partitioning into aggregation-prone intermediates.

**Figure 17.**
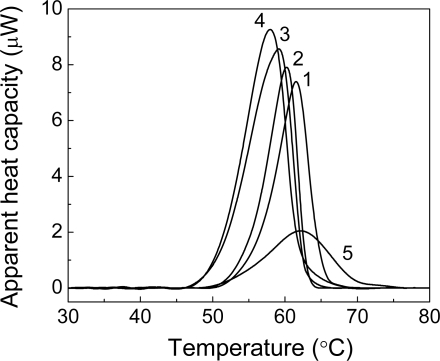
Effect of α-crystallin on thermal denaturation of GAPDH (0.4 mg/mL, 10 mM Na-phosphate buffer, pH 7.5). DSC-profiles were obtained in the absence (curve 1) and in the presence of α-crystallin (curves 2–4). The concentrations of α-crystallin are as follows: (1) 0, (2) 0.1, (3) 0.2 and (4) 0.4 mg/mL. Curve 5 represents the DSC profile for α-crystallin (0.4 mg/mL). The heating rate was 1 °C/min.

It is surprising that in some cases the decrease of the protein stability in the presence of α-crystallin is observed. Khanova *et al.* [121] have studied the effect of α-crystallin on thermal stability of GAPDH using DSC (Figure 17). Thermal unfolding of GAPDH (0.4 mg/mL) is characterized by sharp thermal transition with a maximum at 61.6 °C (curve 1). In the presence of α-crystallin the shift of the maximum position (*T*_max_) towards lower temperatures is observed. The shift is dependent on concentration of α-crystallin. When concentration of α-crystallin is 0.4 mg/mL the value of *T*_max_ decreases to 58.0 °C. These data indicate that stability of GAPDH is markedly reduced in the presence of α-crystallin. The displacement of the maximum position on the DSC profiles of GAPDH towards lower temperatures in the presence of α-crystallin suggests that α-crystallin interacts with the heating-induced intermediates of GAPDH unfolding. The products of heat-induced dissociation of the tetrameric form of GAPDH, namely the dimeric and monomeric forms, may play a role of such unfolding intermediates [94]. It should also be noted that α-crystallin accelerates thermal inactivation of GAPDH at 45 °C (K.A. Markossian, unpublished data). The analogous picture has been observed, when studying the effect of α-crystallin on thermal inactivation and denaturation of mAAT [127].

**Figure 18.**
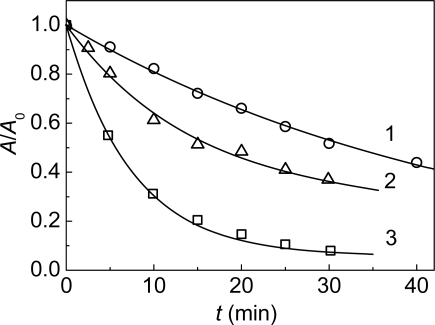
Effect of α-crystallin on the kinetics of thermal inactivation of mAAT (0.1 mg/mL) at 65 **°**C (10 mM Na-phosphate buffer, pH 7.5). The dependences of the relative enzymatic activity (*A/A*_0_) of mAAT. The concentrations of α-crystallin were as follows: (1) 0, (2) 0.05 and (3) 0.1 mg/mL.

The kinetics of thermal inactivation of mAAT (0.1 mg/mL) at 65 °C are shown in Figure 18. This Figure demonstrates the time-course of the decline of the enzymatic activity of mAAT. The time of half-conversion (*t*_0.5_) was found to be 32.7 ± 0.4 min (curve 1). The addition of α-crystallin results in acceleration of the inactivation process. When the concentration of α-crystallin is equal to 0.05 mg/mL, the *t*_0.5_ value decreases to 18.0 ± 0.6 min (curve 2). At the α-crystallin concentration of 0.1 mg/mL the *t*_0.5_ value was found to be 6.3 ± 0.3 min (curve 3).

**Figure 19.**
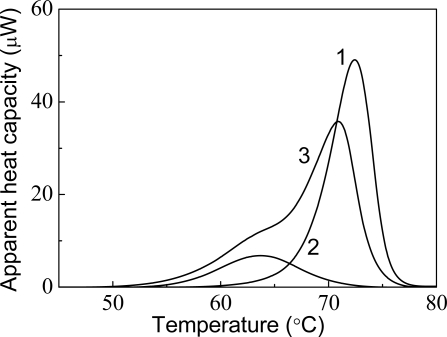
Effect of α-crystallin on thermal denaturation of mAAT (10 mM Na-phosphate buffer, pH 7.5). Curves 1 and 2 correspond to the DSC profiles of mAAT (1 mg/mL) and α-crystallin (1 mg/mL), respectively. Curve 3 corresponds to the DSC profile for the mixture of mAAT (1 mg/mL) and α-crystallin (1 mg/mL). The heating rate was 1 °C/min.

The effect of α-crystallin on the thermostability of mAAT characterized by DSC is shown in Figure 19 [127]. The DSC profile for mAAT (1 mg/mL) has a maximum (*T*_max_) of 72.4 °C (curve 1). The position of a maximum of the DSC profile for α-crystallin (1 mg/mL) corresponds to 63.7 °C (curve 2). The complex shape of the DSC profile for the mixture of mAAT and α-crystallin (curve 3) is due to non-homogeneity of the sample. It is evident that the shoulder at 63.7 °C corresponds to α-crystallin. The *T*_max_ value for the DSC profile under discussion was found to be 70.9 °C. One can assume that such a decrease in the *T*_max_ value is indicative of the reduced stability of mAAT in the presence of α-crystallin. It should be noted that destabilizing effect of α-crystallin has also been demonstrated for *Phb* [97].

**Scheme 1.**
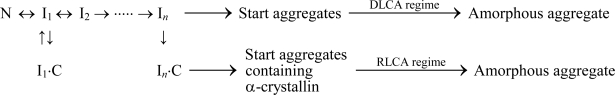
Schematic representation of the possible mechanism explaining the effect of α-crystallin on denaturation of a protein substrate.

Scheme 1 below may serve to explain the effect of α-crystallin on thermal denaturation of a protein substrate. This scheme is a modification of the schematic diagram put forward by Rajaraman *et al.* [122]. In scheme 1 N is the native form of a protein, I_1_ is the early unfolding intermediate and I*_n_* is the late aggregation-prone intermediate. I_1_ ·C and I*_n_* ·C are, respectively, intermediates I_1_ and I*_n_* complexed with α-crystallin. It is assumed that the initial stages of protein unfolding and complexion of α-crystallin with an early unfolding intermediate I_1_ are reversible. Complexation of α-crystallin with the early unfolding intermediate may explain the influence of α-crystallin on denaturation of a protein substrate. One can expect that intermediate I_1_, which is formed as a result of partial unfolding of the native protein molecule, possesses a lower thermostability than the native protein. Protein-protein interactions usually lead to stabilization of the resulting complex. Therefore, one can expect that I_1_ ·C complex is more stable than the I_1_ form. The resulting stability of I_1_ ·C complex may exceed the stability of the native protein form N. In this case α-crystallin decreases the rate of denaturation of the protein substrate (or the rate of inactivation if a protein substrate is an enzyme). However if the decrease in the stability during transition from state N to state I_1_ is sufficiently significant, the resulting stability of the I_1_ ·C complex may be found to be lower than that for the native molecule. In this case α-crystallin will cause destabilization of a protein substrate or, respectively, acceleration of inactivation of an enzyme used as a protein substrate. Thus, Scheme 1 may explain either stabilizing or destabilizing action of α-crystallin, when studying denaturation of a protein substrate (or inactivation of an enzyme).

## 5. Conclusions

One of the functions of molecular chaperones is binding of proteins in unfolded state, which are formed in the course of folding of newly synthesized polypeptide chains or as a result of unfolding of the native proteins under stress conditions. Owing to complexation of unfolded proteins with chaperones, aggregation of proteins in unfolded state is suppressed. The comparative analysis of the anti-aggregation effect of chaperones of different classes is one of the important tasks of the modern biochemistry of chaperones.

An understanding of the mechanism of the anti-aggregation effect of chaperones needs a clarification of the mechanism of protein aggregation. As the DLS studies have shown, the first stage of heat-induced aggregation of proteins is the stage of formation of the starting aggregates. Further growth of the aggregates proceeds as a result of sticking of the starting aggregates and aggregates of higher order. It is evident that the starting aggregates formed in the course of aggregation, resulting in formation of amorphous aggregates, are similar to protofibrils formed in the course of assembly of fibrils.

The anti-aggregation effect of α-crystallin, one of representatives of the family of small heat-shock proteins, is due to diminution of the size of the starting aggregates and decrease in the sticking probability for the colliding particles. A different model may be applicable to chaperones of other classes. For example, chaperonin GroEL, a representative of Hsp60, does not affect the size of the start aggregates, but similarly to α-crystallin, transfers the aggregation process from the diffusion-limited regime to the RLCA regime (K.A. Markossian, unpublished data).

It is significant that the analysis of the dependence of the protein aggregate hydrodynamic radius on time allows revealing proteins that possess their own chaperone-like activity. For example, Markossian *et al.* [128] observed that the dependences of hydrodynamic radius on time for thermal aggregation of yeast alcohol dehydrogenase I (ADH) followed the exponential law. Based on this fact, the authors assumed that ADH might reveal chaperone-like activity. Actually the capability of suppressing aggregation of the protein substrate by ADH was demonstrated in the test-system based on aggregation of UV-irradiated β_L_-crystallin from bovine eye lens [128]. Thus some proteins with well-known biological functions (for example, enzymes) may act as chaperones, enhancing the total chaperone potential of the cell and providing for suppression of unfolded protein aggregation.
